# High attack rate in a large care home outbreak of SARS-CoV-2 BA.2.86, East of England, August 2023

**DOI:** 10.2807/1560-7917.ES.2023.28.39.2300489

**Published:** 2023-09-28

**Authors:** Lucy Reeve, Elise Tessier, Amy Trindall, Nurin Iwani Binti Abdul Aziz, Nick Andrews, Matthias Futschik, Jessica Rayner, Alexis Didier’Serre, Rebecca Hams, Natalie Groves, Eileen Gallagher, Rachael Graham, Beatrix Kele, Katja Hoschler, Tom Fowler, Edward Blandford, Hamid Mahgoub, Jorg Hoffmann, Mary Ramsay, Gavin Dabrera, Meera Chand, Maria Zambon, Ashley Sharp, Ellen Heinsbroek, Jamie Lopez Bernal

**Affiliations:** 1 Field Service East of England, UK Health Security Agency, Cambridge, United Kingdom; 2 COVID-19 Vaccines and Epidemiology Division, UK Health Security Agency, London, United Kingdom; 3 Immunisations and Countermeasures Division, UK Health Security Agency, London, United Kingdom; 4 Public Health and Clinical Oversight, COVID-19 Testing, Clinical and Public Health Division, UK Health Security Agency, London, United Kingdom; 5 East of England Health Protection Team, UK Health Security Agency, Cambridge, United Kingdom; 6 TB, Acute Respiratory, Zoonoses, Emerging infection, and Travel Health Division, UK Health Security Agency, London, United Kingdom; 7 Respiratory Virus Unit, UK Health Security Agency, London, United Kingdom; * These authors contributed equally to this work and share last authorship.

**Keywords:** SARS-CoV-2, BA.2.86 variant, COVID 19 Vaccines, Care Home, Nursing Home, Outbreaks, Epidemiology

## Abstract

We investigated an outbreak of SARS-CoV-2 variant BA.2.86 in an East of England care home. We identified 45 infections (33 residents, 12 staff), among 38 residents and 66 staff. Twenty-nine of 43 PCR swabs were sequenced, all of which were variant BA.2.86. The attack rate among residents was 87%, 19 were symptomatic, and one was hospitalised. Twenty-four days after the outbreak started, no cases were still unwell. Among the 33 resident cases, 29 had been vaccinated 4 months earlier.

By 5 September 2023, outside of this outbreak, 33 cases of infection with severe acute respiratory syndrome coronavirus 2 (SARS-CoV-2) variant BA.2.86 had been reported globally from nine countries [1]. This newly identified variant has a high number of mutations which could confer changes in viral properties including transmissibility and immune evasion [2,3]. On 21 August 2023, the United Kingdom (UK) Health Security Agency (UKHSA) was notified of an outbreak of COVID-19 in a care home in the East of England, with anecdotal reports of a higher attack rate (AR) and severity than previous outbreaks at the home. An outbreak investigation was initiated, and whole genome sequencing confirmed that the causative agent was the SARS-CoV-2 Omicron subvariant BA.2.86 [4]. We present the findings from this outbreak.

## Case definition

A case was defined as a care home resident or staff member with SARS-CoV-2 infection detected by lateral flow device (LFD) or reverse transcription PCR (RT-PCR) between 20 and 28 August 2023. Symptomatic cases reported a variety of cough-cold symptoms, chestiness, lethargy, fatigue, drowsiness or loss of taste or smell.

## Outbreak description

**Figure 1:**
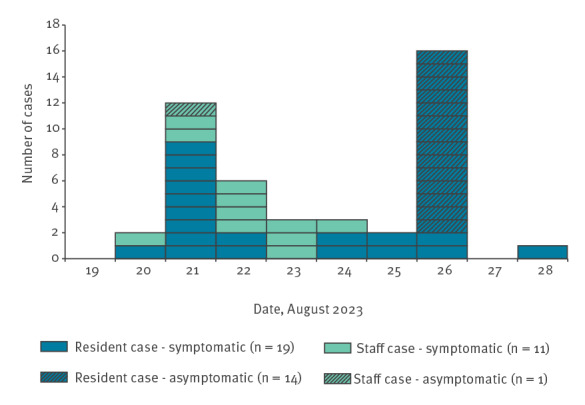
Cases of COVID-19 among care home residents and staff, by symptom onset date if symptomatic or specimen date if asymptomatic, England, August 2023 (n = 45)

An epidemic curve of the outbreak is shown in Figure 1. The index case was a symptomatic resident who tested positive for SARS-CoV-2 by LFD on 20 August 2023. By 21 August, three staff members and 10 residents were symptomatic. Following existing guidance to test a sample of symptomatic persons [5], the first seven symptomatic residents were tested by LFD: all were positive. At this time, four staff members also tested positive by LFD, one of whom was asymptomatic.

Date of mass swabbing for PCR: 26 August 2023.

Given the rapid spread and concerns from the general practitioner (GP) that symptoms were qualitatively worse than observed in previous outbreaks, the care home management team requested UKHSA support. In total, 17 people were LFD-positive (seven residents and 10 staff). Mass swabbing for PCR was done on 26 August for all residents (n = 38) and for all symptomatic or LFD-positive staff members (n = 12 staff eligible; n = 10 swabs submitted).

Overall, 33 of 38 residents tested positive by LFD or PCR (AR = 87%), of whom 19 were symptomatic (Figure 1, Table 1). Four resident cases recorded low oxygen saturation and nine required healthcare attendance (GP consultation or ambulance attendance) including one resident who was hospitalised overnight. Two resident cases died within 16 days of symptom onset; these deaths were considered unrelated to COVID-19 and occurred after their COVID-19 symptoms resolved. No cases were eligible for antiviral treatment, but two residents were prescribed antibiotics for suspected secondary infections. By 13 September (24 days after the outbreak started), 17 symptomatic residents had recovered, with none still unwell. Thirteen symptomatic residents had recovered within 14 days of symptom onset.

**Table 1:** Description of cases of COVID-19 among care home residents and staff, England, August 2023 (n = 45)

	Resident cases		Staff cases^a^	
	n	%	n	%
**Total**	33		12	
**Demographics**				
Mean age in years	87		31	
Sex^b^ (female)	26	79	10	83
Sex^b^ (male)	7	21	2	17
**Testing method**				
PCR- and LFD-positive	7	21	8	67
PCR-positive only (not tested by LFD)	26	79	2	17
LFD-positive only (not tested by PCR)	0	0	2	17
**Symptoms**				
Symptomatic	19	58	11	92
Asymptomatic	14	42	1	8
**Clinical features of symptomatic cases** ^c^				
Low oxygen saturations recorded	4	12	0	0
Required any healthcare attendance^d^	9	27	0	0
Received antibiotics for secondary infection	2	6	0	0
Hospitalisation	1	3	0	0
**Outcome**				
Recovered	31	94	12	100
Still unwell (24 days after start of outbreak)	0	0	0	0
Died of other cause	2	6	0	0
**Time since last vaccination dose**				
4 months	29	88	0	0
10–11 months	2	6	3	25
> 20 months	1	3	7	58
Never vaccinated	0	0	0	0
Unknown time since last vaccination dose	1	3	2	17

LFD: lateral flow device.

^a^ Note that only staff who were symptomatic or LFD-positive were tested.

^b^ Sex was collected as male, female, unknown, or prefer not to say (no missing values).

^c^ Clinical features categories are not mutually exclusive.

^d^ Defined as general practitioner consultation, ambulance attendance or hospitalisation.

All 11 symptomatic staff were tested and one asymptomatic. Overall, 12 of the total staff of 66 tested positive by LFD and/or PCR (AR = 18%) and no tested staff were negative. Ten symptomatic staff had recovered within 14 days of symptom onset.

## Control measures

The incident management team were satisfied by the care home’s implementation of infection prevention and control measures according to current UKHSA guidance [5]. Staff cases isolated from work and returned after 5 days or once recovered. Staff cohorting of resident care continued, with personal protective equipment (PPE) available in each room. Enhanced cleaning was undertaken in communal areas, with touch-point cleaning three times daily. Enhanced cleaning was undertaken in resident rooms, equipped with their own hand basin and toilet, at the end of the resident's isolation period. Linens from positive residents were washed separately. Imminent care home admissions were cancelled, and visiting was limited to one person at a time, with hand washing and PPE donned immediately upon entry to the care home.

## Vaccination status

Self-reported vaccination statuses were verified by linkage to the national immunisation monitoring system for all residents and for 10 of 12 staff cases, enabling calculation of the time since their last dose [6]. Thirty-four of 38 residents had received a booster dose of COVID-19 vaccine VidPrevtyn Beta (Sanofi Pasteur) approximately 4 months before the outbreak, a recombinant spike vaccine based on the Beta variant, and the main vaccine used early in the national vaccination campaign in spring 2023 [7]. Of the five residents who tested negative, none had symptoms, and all had received a booster vaccination in spring 2023. All 12 staff cases had received at least one COVID-19 vaccine (n = 2 self-reported); three of 12 had received a dose as part of the booster campaign in autumn 2022 ca 10 months before the outbreak.

The infection AR among residents vaccinated 4 months before the outbreak was 29 of 34 (85%; 95% confidence interval (CI): 69–95) compared with four of four (100%; 95% CI: 40–100) in those with no evidence of vaccination at this time (Table 2). This difference is not significant (Fisher’s exact test; p = 1.00) and gives an estimated effectiveness of 15% (95% CI: −47 to 32). When considering symptomatic infection as an outcome, including asymptomatic persons as non-cases, the AR in those vaccinated in spring was 16 of 34 (47%; 95% CI: 30–65) and in those not recently vaccinated it was three of four (75%; 95% CI: 19–99), resulting in an effectiveness against symptomatic infection of 37% (95% CI: −22 to 68; p = 0.60) (Table 2).

**Table 2:** Assessment of vaccine effectiveness for vaccination 4 months previously in care home residents against infection (all positive) and against symptomatic infection, England, August 2023 (n = 38)

Case definition	Vaccinated 4 months previously	Cases	Total	% AR (95% CI)	VE (95% CI)^a^	p value^b^
All positive	Yes	29	34	85 (69–95)	15% (−47 to 32)	1.00
	No	4	4	100 (40–100)		
All symptomatic positive	Yes	16	34	47 (30–65)	37% (−22 to 68)	0.60
	No	3	4	75 (19–99)		

AR: attack rate; CI: confidence interval; VE: vaccine effectiveness.

^a^ VE = 1 − attack rate in vaccinated/attack rate in unvaccinated. Note that due to the 100% attack rate in unvaccinated residents, the 95% CI for all positive cases was based on adding a count of 1 to the total of vaccinated cases, vaccinated non-cases, unvaccinated cases and unvaccinated non-cases.

^b^ Fisher's exact test.

## Genomic analysis

**Figure 2:**
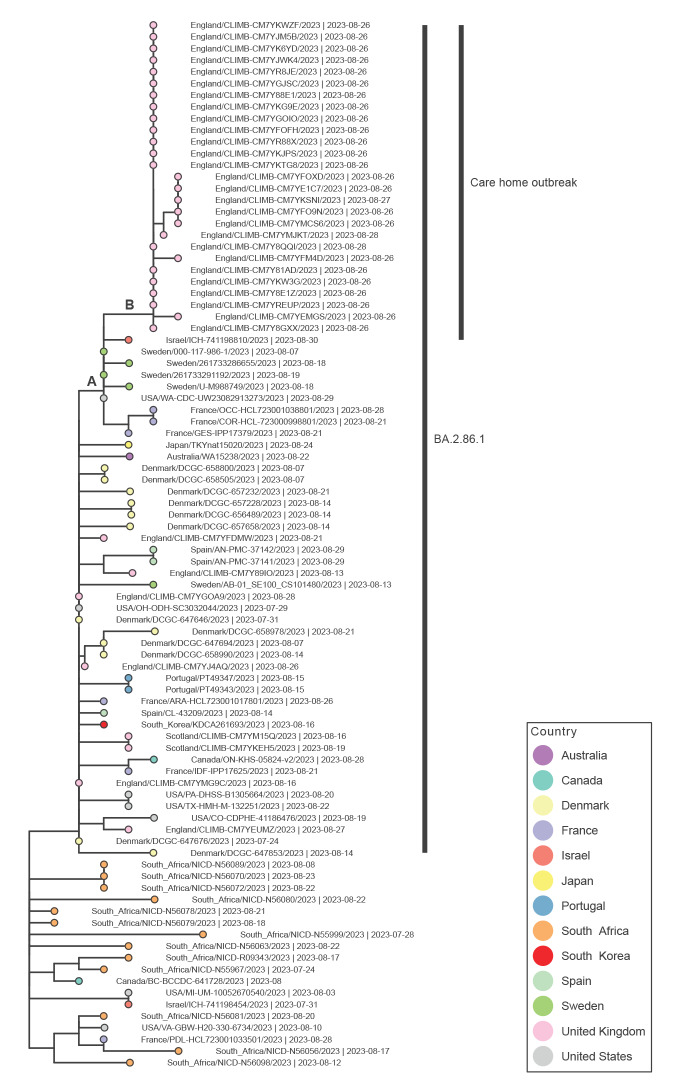
Phylogenetic tree of SARS-CoV-2 BA.2.86 genomes generated from cases in this cluster that were of sufficient quality, England, September 2023 (n = 27) and sequences available in GISAID at 15:00 7 September 2023

All positive PCR tests were sent for whole genome sequencing using the Artic protocol (primer pool version 5.3.2) at UKHSA Colindale Virus Reference Department [8]. In total, 32 of 43 samples were suitable for sequencing (quantification cycle (Cq) ≤ 32). The results for 29 of the 32 samples, (22 from residents and seven from staff) were confirmed as lineage BA.2.86 with a genome coverage of 93.9–99.9%, while three samples had low genome coverage (53–77%) [9]. Figure 2 shows a phylogenetic tree containing all BA.2.86 sequences available in GISAID [10] at 15:00 on 7 September 2023 (available at http://dx.doi.org/10.55876/gis8.230908dx) and 27 of the 29 sequences generated for the confirmed BA.2.86 infections in this cluster [11].

The sequences from this cluster formed a unique clade (labelled as Care home outbreak in Figure 2) within the larger clade designated as lineage BA.2.86.1 (indicated in Figure 2) and did not cluster with any other cases from the UK identified to date. The clade is separated from other BA.2.86 sequences (marked B in Figure 2) by two additional mutations: a synonymous change in *nsp2*, 2527G>A; and a non-synonymous change in *nsp3*, P153L. The clade is most closely related to sequences from Sweden and France (marked A in Figure 2), and the sequences from this cluster share an additional non-synonymous mutation (*nsp2*: Y441C) with these sequences compared with other BA.2.86 sequences. Additional details on genetic mutations within this clade are made available in the Supplement.

## Lateral flow device sensitivity

Twelve of 15 LFDs were of the same brand of test kit (Orient Gene), for which previously a sensitivity of 72% (95% CI: 67–76%) was observed during a period of predominant Omicron infections [12]. Given complete concordance of our LFD and PCR results, the performance of LFDs was assessed, with 100% sensitivity and a lower CI of 72%. Non-inferiority to the previous performance can be declared at a 0.1% significance level, and the probability of reduced sensitivity to detect BA.2.86 is small (5%). We provide additional detail on our statistical methods in the Supplement.

## Discussion

At the time of the outbreak investigation, data suggested established community transmission of BA.2.86 due to the detection of multiple sporadic cases in different regions of the UK [11]. This is the first reported care home outbreak with SARS-CoV-2 BA.2.86; our analyses provide additional understanding of AR, severity, vaccine effectiveness (VE) and LFD sensitivity compared with the occurrence of BA.2.86 in Denmark [3].

Cases were initially detected in the care home described here by LFD tests: symptomatic residents were tested according to current guidance, and while staff testing is no longer required, 10 staff self-tested of their own accord [5]. The majority of cases were female, which reflects the sex distribution of the care home residents (82% female), the sex distribution is unknown for staff members. While there is some epidemiological evidence of the introduction of COVID-19 into this care home, there were no samples available for retrospective confirmatory testing, nor is information available on possible infections among visitors during the outbreak.

The AR in this setting was substantially higher than that seen in nine UK care homes that experienced outbreaks in December 2021 to January 2022; the early period in which the Omicron variant circulated in the UK (23%) [13], although it was similar to one care home outbreak during early circulation of the Delta variant (82%) [14]. This suggests high transmissibility, although enhanced testing was undertaken in this outbreak specifically because of the reported high number of cases. Therefore, the AR observed is subject to selection bias and may not be typical of all care home outbreaks involving the BA.2.86 variant as the AR in care homes may vary due to a range of factors [2].

The hospitalisation rate among residents was lower than that reported in care home outbreaks during early circulation of the Omicron variant in December 2021 to January 2022 (11%) [13] and Delta variants (17%) [14]. Although the care home reported that the symptoms had a greater impact on the day-to-day life of residents and staff compared with their previous outbreaks, it is reassuring that there was no notable increase in the most severe outcomes of hospitalisation and death associated with BA.2.86 [13,14]. Nonetheless, hospitalisation rates in care homes are very context-dependent, therefore these comparisons may not be generalisable to other settings.

The high vaccination rate in the care home and lack of substantial vaccine effect against infection in this outbreak 4 months after a booster dose suggests notable immune evasion. This is not unexpected as we have seen rapid waning of VE against infection and mild disease with other vaccines and previous Omicron subvariants [15]. While the numbers were too small for any precise assessment of VE, the point estimate against symptomatic infection 4 months after a dose was similar to that observed with previous Omicron variants [16]. With previous variants, VE against severe disease resulting in hospitalisation has been considerably higher than against infection, and this is likely to also be the case with BA.2.86 [17].

Statistical assessment indicated that the LFD predominantly used in this outbreak had non-inferior sensitivity to detect BA.2.86 compared with previous SARS-CoV-2 Omicron strains [12]. However, LFD performance tends to be strongly associated with viral load, and stratified analysis of a larger sample would provide a more unbiased comparison.

## Conclusion

This outbreak has provided useful epidemiological insight into the characteristics of the SARS-CoV-2 BA.2.86 variant. While the findings may not be generalisable to other settings, we see evidence of limited VE against infection 4 months after vaccination, high transmissibility of the variant but no suggestion of increased severity.

## Supplementary Data

145000 Supplement
