# Supplementary material for: High attack rate in a large care home outbreak of SARS-CoV-2 BA.2.86, East of England, August 2023
Source: Euro Surveill. 2023 Sep 28;28(39):2300489. doi: 10.2807/1560-7917.ES.2023.28.39.2300489 (PMC10540514; doi:10.2807/1560-7917.ES.2023.28.39.2300489)
Supplement: Supplement [file 23-00489_REEVE_Supplement.pdf]

Supplementary Materials

This supplementary material is hosted by *Eurosurveillance* as supporting information alongside the article '*High attack rate in a large care home outbreak of SARS-CoV-2 BA.2.86, East of England, August 2023*', on behalf of the authors, who remain responsible for the accuracy and appropriateness of the content. The same standards for ethics, copyright, attributions and permissions as for the article apply. Supplements are not edited by *Eurosurveillance* and the journal is not responsible for the maintenance of any links or email addresses provided therein.

Supplement S1, Statistical assessment of lateral flow device sensitivity in the outbreak of BA.2.86 in a care home in the East of England:

To assess whether we can stringently exclude any diminishing lateral flow device (LFD) performance for BA.2.86 based on the limited number of available LFD results, a statistical assessment was undertaken. For 15 LFDs used as self-test by symptomatic individuals, positive results were reported. All positive LFD results were confirmed by positive reverse transcription-polymerase chain reaction (RT-PCR) tests. Of the 15 LFDs, 12 were of type Orient Gene (OG). For this type of LFDs applied as self-test by symptomatic individuals within the time period from 8/01/2022 to 21/03/2022, a sensitivity of 71.8% (95% Confidence Interval (CI): 67.4% - 76.0%) was observed (1). The dominant SARS-Cov-2 strains during that period were B.1.1.529 (Omicron), BA.1.1 (Omicron) and BA.2 (Omicron). 95% CIs were calculated using the Agresti-Caffo formula which provides a higher coverage probability for extreme proportions such as 100% (2). The 95% CI for the sensitivity based on 95% CI based on the 12 OG LFDs was from 71.8% to 100.0%.

To assess statistically the possibility of inferior sensitivity of OG LFDs for BA.2.86, non-inferiority was tested against the baseline sensitivity of 71.8% with a pre-defined margin δ of 5% at a significance level α of 0.001 (3). Thus, the lower limit of the $(1–2\alpha) \times 100\%$ CI was compared to a threshold of 66.8% (= 71.8% - δ). Calculation of the 99.8% CI for the difference between sensitivities estimated for the outbreak and the baseline OG LFD sensitivity showed that the lower limit of the CI is above the threshold and non-inferiority can be declared at a 0.1% significance level.

Finally, the probability that the sensitivity to detect BA.2.86 is below our baseline was determined as 5% by variation of the width of the CI from 80% to 99.99%.

References:

1. UK Health Security Agency. Evaluation of lateral flow device performance within the National Testing Programme 2023 [Available from: https://assets.publishing.service.gov.uk/government/uploads/system/uploads/attachment_data/file/1121707/LFD_Performance_within_the_NTP_Report.pdf.
2. Agresti A, Caffo B. Simple and effective confidence intervals for proportions and differences of proportions result from adding two successes and two failures. Am Stat. 2000;54(4):280-8.
3. Walker E, Nowacki AS. Understanding equivalence and noninferiority testing. J Gen Intern Med. 2011;26(2):192-6.

Supplement S2, Details of additional genetic mutations seen in cluster BA.2.86.1:

Two sequences from this cluster each have a single additional mutation not seen in other sequences from this cluster: nsp2 A318V in CLIMB-CM7YEMGS; and nsp2 F406C in CLIMB-CM7YFM4D. Five sequences share an additional Spike mutation (D1139N) not seen in other sequences from this cluster. One additional sequence, CLIMB-CM7YMJKT contains a mixed base at this position.
